# Contributions of the international plant science community to the fight against infectious diseases in humans—part 2: Affordable drugs in edible plants for endemic and re‐emerging diseases

**DOI:** 10.1111/pbi.13658

**Published:** 2021-07-19

**Authors:** Wenshu He, Can Baysal, Maria Lobato Gómez, Xin Huang, Derry Alvarez, Changfu Zhu, Victoria Armario‐Najera, Aamaya Blanco Perera, Pedro Cerda Bennaser, Andrea Saba‐Mayoral, Guillermo Sobrino‐Mengual, Ashwin Vargheese, Rita Abranches, Isabel Alexandra Abreu, Shanmugaraj Balamurugan, Ralph Bock, Johannes F. Buyel, Nicolau B. da Cunha, Henry Daniell, Roland Faller, André Folgado, Iyappan Gowtham, Suvi T. Häkkinen, Shashi Kumar, Ramalingam Sathish Kumar, Cristiano Lacorte, George P. Lomonossoff, Ines M. Luís, Julian K.‐C. Ma, Karen A. McDonald, Andre Murad, Somen Nandi, Barry O’Keef, Subramanian Parthiban, Mathew J. Paul, Daniel Ponndorf, Elibio Rech, Julio C.M. Rodrigues, Stephanie Ruf, Stefan Schillberg, Jennifer Schwestka, Priya S. Shah, Rahul Singh, Eva Stoger, Richard M. Twyman, Inchakalody P. Varghese, Giovanni R. Vianna, Gina Webster, Ruud H. P. Wilbers, Paul Christou, Kirsi‐Marja Oksman‐Caldentey, Teresa Capell

**Affiliations:** ^1^ Department of Crop and Forest Sciences University of Lleida‐Agrotecnio CERCA Center Lleida Spain; ^2^ Instituto de Tecnologia Química e Biológica António Xavier Universidade Nova de Lisboa Oeiras Portugal; ^3^ Plant Genetic Engineering Laboratory Department of Biotechnology Bharathiar University Tamil Nadu India; ^4^ Max Planck Institute of Molecular Plant Physiology Potsdam‐Golm Germany; ^5^ Fraunhofer Institute for Molecular Biology and Applied Ecology IME Aachen Germany; ^6^ Institute for Molecular Biotechnology RWTH Aachen University Aachen Germany; ^7^ Centro de Análise Proteômicas e Bioquímicas de Brasília Universidade Católica de Brasília Brasília Brazil; ^8^ School of Dental Medicine University of Pennsylvania Philadelphia PA USA; ^9^ Department of Chemical Engineering University of California, Davis Davis CA USA; ^10^ Industrial Biotechnology and Food Solutions VTT Technical Research Centre of Finland Ltd Espoo Finland; ^11^ International Centre for Genetic Engineering and Biotechnology New Delhi India; ^12^ Brazilian Agriculture Research Corporation Embrapa Genetic Resources and Biotechnology and National Institute of Science and Technology Synthetic in Biology, Parque Estação Biológica Brasilia Brazil; ^13^ Department of Biological Chemistry John Innes Centre Norwich Research Park, Norwich UK; ^14^ Institute for Infection and Immunity St. George’s University of London London UK; ^15^ Global HealthShare Initiative University of California, Davis Davis CA USA; ^16^ Division of Cancer Treatment and Diagnosis Molecular Targets Program Center for Cancer Research National Cancer Institute, and Natural Products Branch, Developmental Therapeutics Program National Cancer Institute, NIH Frederick MD USA; ^17^ Institute for Phytopathology Justus‐Liebig‐University Giessen Giessen Germany; ^18^ Institute of Plant Biotechnology and Cell Biology University of Natural Resources and Life Sciences Vienna Austria; ^19^ Department of Microbiology and Molecular Genetics University of California, Davis Davis CA USA; ^20^ TRM Ltd Scarborough UK; ^21^ Laboratory of Nematology Plant Sciences Group Wageningen University and Research Wageningen The Netherlands; ^22^ ICREA Catalan Institute for Research and Advanced Studies Barcelona Spain

**Keywords:** molecular farming, plant‐made pharmaceuticals, oral delivery, endemic disease, re‐emerging disease

## Abstract

The fight against infectious diseases often focuses on epidemics and pandemics, which demand urgent resources and command attention from the health authorities and media. However, the vast majority of deaths caused by infectious diseases occur in endemic zones, particularly in developing countries, placing a disproportionate burden on underfunded health systems and often requiring international interventions. The provision of vaccines and other biologics is hampered not only by the high cost and limited scalability of traditional manufacturing platforms based on microbial and animal cells, but also by challenges caused by distribution and storage, particularly in regions without a complete cold chain. In this review article, we consider the potential of molecular farming to address the challenges of endemic and re‐emerging diseases, focusing on edible plants for the development of oral drugs. Key recent developments in this field include successful clinical trials based on orally delivered dried leaves of *Artemisia annua* against malarial parasite strains resistant to artemisinin combination therapy, the ability to produce clinical‐grade protein drugs in leaves to treat infectious diseases and the long‐term storage of protein drugs in dried leaves at ambient temperatures. Recent FDA approval of the first orally delivered protein drug encapsulated in plant cells to treat peanut allergy has opened the door for the development of affordable oral drugs that can be manufactured and distributed in remote areas without cold storage infrastructure and that eliminate the need for expensive purification steps and sterile delivery by injection.

## Introduction

Infectious diseases can be classified according to prevalence, with **rare diseases** affecting a small proportion of the population (less than one in 1500–2500 people, depending on the jurisdiction) either due to a stable low transmission rate or because the disease is **emerging** or **re‐emerging** (resurgent) and the incidence is low but increasing. Infectious diseases can also be divided into categories based on their epidemiology and are often described as epidemic or pandemic (discussed in our sister article in this issue Lobato Gómez *et al*., 2021) or endemic in a given region.

An **endemic disease** is prevalent in a particular geographic area due to sustained local transmission, but broader dissemination is prevented by factors such as vector range/habitat (e.g. malaria is restricted by the natural range of mosquito vectors) or technologies (e.g. helminth infections are restricted to areas that lack water purification). A **hyperendemic disease** has a high level of incidence in an endemic area and can even trigger local epidemics, in which a more serious outbreak is sudden and unexpected (CDC, 2020b). When an endemic disease affects almost all individuals in an endemic area, it is described as **holoendemic**, although the symptoms may differ in severity through the population, as seen for malaria (WHO, 2016). Travel can spread endemic diseases beyond their normal range (Wilson, 2005). However, the establishment of such diseases in new ranges is limited by the factors described above and by the strict imposition of vaccination programmes for travellers (CDC, 2020a).

This review article assesses the use of plant biotechnology as a means to tackle endemic diseases, focusing on the use of plants as bioreactors for the production of pharmaceutical products including protein drugs, vaccines, antibodies and antivirals for established endemic diseases as well as neglected and re‐emerging diseases. The diseases covered by the article are summarized in Table 1, with more details on the incidence, prevalence and burden associated with these diseases provided in Table S1.

**Table 1:** Classification of infectious diseases based on their epidemiology, showing the number of people affected in a specific time and place. The fatality rate was calculated by dividing the number of deaths by the total number of identified cases in the specific time and place. References are listed in Table S1

Classification	Disease	Number of people affected	Fatality rate
Endemic diseases	Dengue fever	4.2 million (total of global cases, 2019)	1%
	West Nile fever	2645 (USA, 2018)	6.2%
	Yellow fever	2399 (December 2015–February 2018)	29%
	Rabies	59,000/year (total of global cases estimate)	100% (once symptoms appear)
	Malaria	229 million (total of global cases, 2019)	0.18%
	Tuberculosis	7.6 million (total of global cases, 2018)	19.7%
	Helminth diseases	1.5 billion (total of global cases, 2019)	< 1%
Re‐emerging or rare/neglected diseases	Cholera	131,121 (total of global cases, 2016)	1.8%
	Measles	9.7 million (total of global cases, 2015)	1.4%
	CCHF	>1000 people/year (south‐eastern Europe)	32.4%
	Polio	Varies from <100 to ˜1000 (Pakistan, Afghanistan)	0.1%

Abbreviations: CCHF = Crimean–Congo haemorrhagic fever.

## Plants for the mucosal delivery of pharmaceuticals

Plant biotechnology has multiple roles in the fight against infectious diseases, from the provision of emergency testing infrastructure (Webb *et al*., 2020) to the manufacture of small‐molecule drugs, biologics (vaccines and therapeutics) and diagnostic reagents (Capell *et al*., 2020; McDonald and Holtz, 2020; Rosales‐Mendoza, 2020; Tusé *et al*., 2020). The utilization of plants to provide injectable drugs for the treatment of epidemic and pandemic diseases is reviewed in detail in our sister article in this issue (Lobato Gómez *et al*., 2021). However, the most dangerous human pathogens cause respiratory or gastroenteric diseases and enter the body through mucosal surfaces. In addition to inducing systemic immunity, it is therefore advantageous to achieve passive or active mucosal immunity via oral or nasal administration in order to prevent infection and decrease transmission. Indeed, mucosal and systemic immune responses often differ both qualitatively and in terms of potency. However, a major challenge for mucosal vaccines and therapeutics is the need for them to withstand degradation, especially following oral delivery. To ensure that the active components remain intact upon arrival at their effector site, they must be fortified to prevent degradation. Infections of the mucosal surfaces of the lungs and upper airways can also be treated with surface‐acting antimicrobials that do not require uptake, such as bacteriophage‐derived endolysins (Bock and Warzecha, 2010; Oey *et al*., 2009a,b).

## Oral delivery of proteins bioencapsulated in plant cells

Plant cell walls contain polysaccharides with β(1,4) and β(1,6) linkages that resist hydrolysis by enzymes in the mammalian upper digestive tract, thus protecting protein drugs and vaccine antigens from the acids and enzymes in the stomach. The survival of recombinant proteins encapsulated in plant cells after passing through the stomach has been demonstrated for reporter proteins (Xiao *et al*., 2016) as well as many therapeutic proteins (Daniell *et al*., 2016, 2019a, 2021; Kwon and Daniell, 2016). The plant cell wall is subsequently digested by enzymes produced by commensal bacteria in the small intestine, where most absorption occurs (Kumar *et al*., 2020). The next major challenge is to ensure that recombinant proteins released from plant cells cross the gut epithelium (Rosales‐Mendoza and Salazar‐Gonzalez, 2014). Unless the proteins naturally contain epithelial receptor‐binding ligands or possess the ability to cross using another mechanism, fusion tags are required comprising receptor ligands or cell‐penetrating peptides, allowing orally administered proteins to reach targets such as the immune system or to enter the systemic circulation. This is often achieved using the non‐toxic cholera toxin B subunit (CTB), which binds specifically to GM1 ganglioside receptors enriched in the membrane lipid rafts of intestinal epithelial cells and travels in a retrograde direction through the *trans*‐Golgi network into the endoplasmic reticulum.

The mucosal area of the human small intestine is ˜30 m^2^ (Helander and Fandriks, 2014). Up to 15,000 CTB molecules can bind to each intestinal epithelial cell and the GM1 receptor is rapidly turned over on the cell surface (Fishman *et al*., 1983). This facilitates the efficient uptake of vaccine antigens and other proteins. The efficiency of CTB‐mediated transmucosal delivery allows drug doses to be reduced by 500‐fold compared to proteins without a CTB tag (Petersen *et al*., 2003). The CTB allows delivery to the immune system or circulation, but the presence of a protein transduction domain (PTD) ensures delivery to the circulatory system whereas the presence of a dendritic cell peptide ensures delivery solely to dendritic cells (Xiao *et al*., 2016). When fusion proteins cross the epithelium, tags are removed by the furin protease present in most cell types if an engineered cleavage site is present (Daniell *et al*., 2016, 2019a; Kwon and Daniell, 2016; Xiao *et al*., 2016). In addition to these *in vivo* studies, cell‐based models have shown that fluorescent protein bodies are taken up by immune cells, potentially into late endosomes, where antigen processing takes place (Schwestka *et al*., 2020; Snapper, 2018;).

Not all oral drugs are designed to cross the epithelial lining but may instead be intended to work in the lumen or in the mucosal layer. For example, successful passive immunization has been demonstrated by feeding chickens with pea seeds expressing antibodies against gut parasites (Zimmermann *et al*., 2009) and feeding piglets with soybean containing monomeric IgA to prevent enterotoxigenic *Escherichia coli* (F4‐ETEC) infection (Virdi *et al*., 2019).

## The importance of protein expression levels and compartmentalization within plant cells

**Figure 1:**
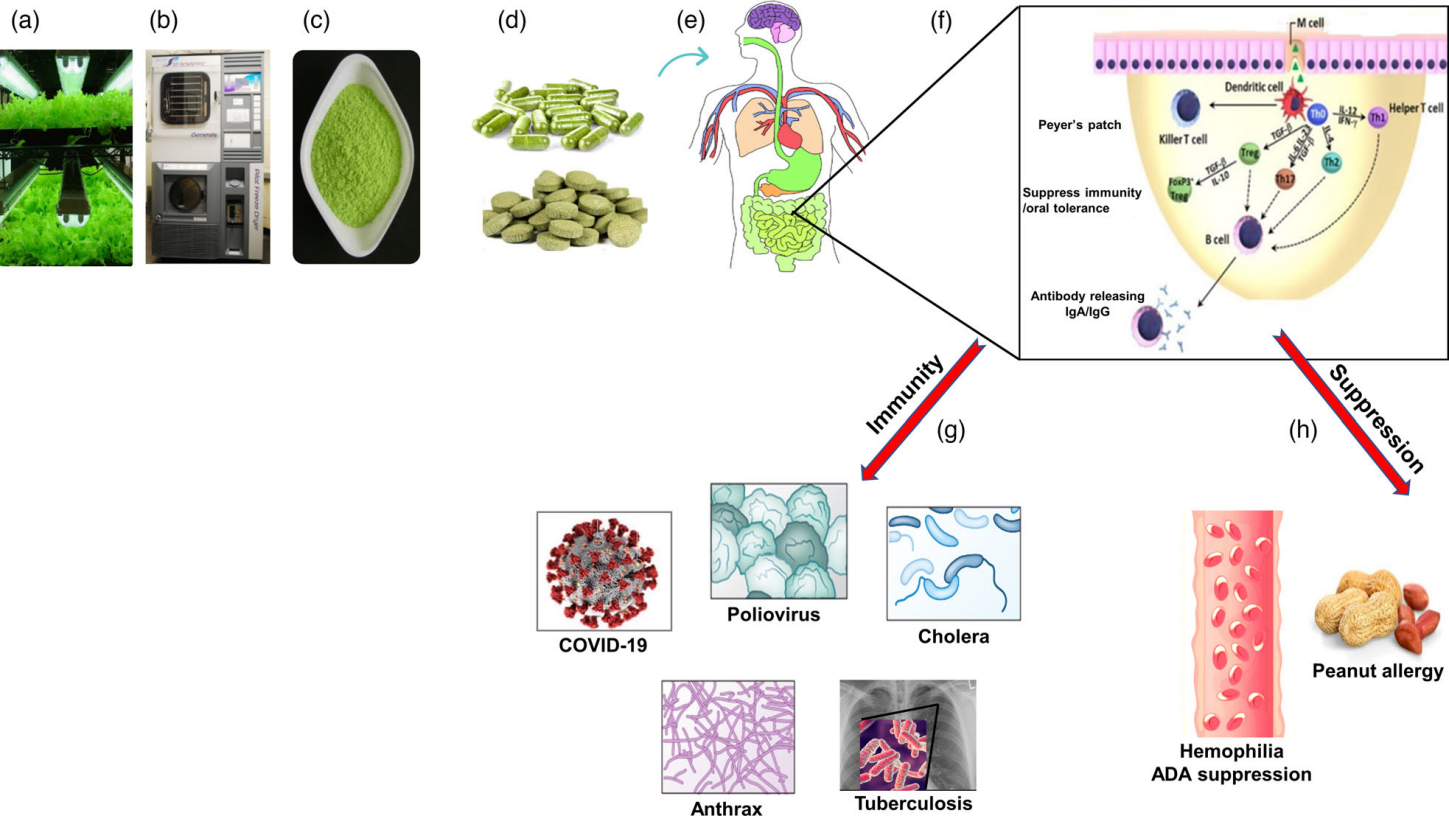
Mechanism of oral drug delivery and examples of chloroplast therapeutics and booster vaccines. (a) The cGMP growing facility for lettuce leaf biomass production. (b) The lyophilizer that dehydrates lettuce biomass through optimized programming for freeze‐drying. (c) Optimized grinding to maintain the intactness of plant cells. (d) The capsule/gum preparation maintaining antigen stability. (e) The oral delivery of proteins bioencapsulated in plant cells. (f) Mechanism of immune suppression/tolerance or conferring immunity. GM1 ganglioside receptors on intestinal epithelial cells facilitate uptake CTB‐fused proteins. DCs are antigen‐presenting cells, induce antigen‐specific T and B cells. The IFN‐γ and Th2 cytokines (IL‐4, IL‐10) are critical for cell‐mediated and humoral immunity. After TGF‐β production, FoxP3^+^ T_reg_ cells are induced by DCs. The immune tolerance via induction and maintenance of FoxP3^+^ T_reg_ cells is mediated by TGF‐β. (g) Examples of potential chloroplast‐derived booster vaccines against viral (polio, COVID‐19) and bacterial (anthrax, cholera, tuberculosis) diseases. (h) Oral tolerance induction and immune suppression in haemophilia and the prevention of peanut allergy.

One of the major limitations of molecular farming for oral drug delivery is the requirement for high yields of the recombinant protein in edible plant tissues. The administration of edible plant tissues removes the opportunity for purification or concentration, but these processes also add to the costs of production and introduce the requirement for a cold chain (Kashima *et al*., 2016). To address this issue, several approaches have been developed to increase the quantity of recombinant proteins within edible plant tissues, including the expression of proteins in chloroplasts, targeting proteins to storage organelles, enhancing protein stability in starch granules and the production of self‐assembling virus‐like particles (VLPs) (Hofbauer *et al*., 2016; Khan *et al*., 2012; Marsian and Lomonossoff, 2016; Schwestka *et al*., 2020; Takagi *et al*., 2008; Whitehead *et al*., 2014). Significant progress has been made using the lettuce chloroplast system (Figure 1a‐e) because each cell typically contains up to 10,000 chloroplast genomes, resulting in expression levels of up to 70% total leaf protein (Daniell *et al*., 2016, 2019a; Ruhlman *et al*., 2010;). Thin lettuce leaves facilitate rapid lyophilization and the antigens remain stable for years in the dry plant material (Daniell *et al*., 2020, 2021; Herzog *et al*., 2017; Park *et al*., 2020; Su *et al*., 2015;).

Many protein drugs have now been expressed in plastids, ranging from small regulatory proteins such as Ang1‐7 (Shenoy *et al*., 2014; Shil *et al*., 2014;), exendin (Kwon *et al*., 2013), antimicrobial peptides (DeGray *et al*., 2001; Lee *et al*., 2011b), insulin (Boyhan and Daniell, 2011) and growth hormones (Daniell *et al*., 2009; Park *et al*., 2020; Staub *et al*., 2000) to very large proteins (Daniell *et al*., 2020, 2021; Kwon *et al*., 2016, 2018; Sherman *et al*., 2014; Su *et al*., 2015). Plastid‐derived vaccine antigens against several diseases have induced high antibody titres, conferring mucosal and/or systemic immunity and in some cases protection against multiple pathogen challenges (Figure 1f,g), including tetanus (Tregoning *et al*., 2003, 2005), cholera (Daniell *et al*., 2001; Davoodi‐Semiromi *et al*., 2010;), bubonic plague (Arlen *et al*., 2008), tuberculosis (Lakshmi *et al*., 2013), anthrax (Koya *et al*., 2005; Ruhlman *et al*., 2010; Watson *et al*., 2004;), malaria (Davoodi‐Semiromi *et al*., 2010), dengue (van Eerde *et al*., 2019; Kanagaraj *et al*., 2011;) and polio (Chan *et al*., 2016; Daniell *et al*., 2019b; Xiao and Daniell, 2017). There is also some potential for the treatment of COVID‐19 (Daniell, 2020; Daniell *et al*., 2021;). More recent iterations of this technology can generate vaccine‐producing marker‐free lettuce plants (Daniell *et al*., 2019c, 2020; Kumari *et al*., 2019; Park *et al*., 2020).

## Oral delivery of proteins to confer immunity or tolerance

The oral delivery of vaccine antigens in edible plant tissues has been explored for decades, with the first clinical trials taking place more than 20 years ago (Tacket *et al*., 1998, 2000). However, the gut immune system works towards immune suppression and the induction of oral tolerance rather than immunity (Figure 1f,h). Oral drug delivery is therefore ideal for the development of tolerance to peanut allergy via the gut immune system. This recently resulted in the approval of Palforzia, an oral drug based on endogenous Arah proteins in peanut cells (Berglund *et al*., 2017; Du Toit *et al*., 2015; Tilles and Petroni, 2018; Vickery *et al*., 2018). The same principle has been used to produce recombinant allergy vaccines in rice seeds for allergen‐specific oral immunotherapy (Takaiwa *et al*., 2015). A peptide vaccine‐containing seven linked human‐predominant T‐cell epitopes derived from Japanese cedar pollen allergens were also produced in rice (Saito *et al*., 2020), and the efficacy and safety of the transgenic rice grains were confirmed after oral administration to JC pollinosis patients (Endo *et al*., 2021).

The ability of the gut immune system to switch from tolerance to immunity is induced by the administration of adjuvants, which may be supplied orally (along with the antigen) or by injection. The failure of previous vaccine trials may in some cases therefore reflect the lack of oral adjuvants to prime the gut immune system (Daniell *et al*., 2019b). Orally delivered antigens bioencapsulated in plant cells elicit high levels of systemic (IgG1) and mucosal (IgA) antibodies but do not confer protection against virus challenge without priming by injected antigens and adjuvants (Chan *et al*., 2016; Daniell *et al*., 2019a; Xiao and Daniell, 2017). Nevertheless, the COVID‐19 pandemic clearly illustrates the need for cold chain‐free booster vaccines, and antigens made in plant cells could thus play an important role by producing booster vaccines rather than primary vaccines. The SARS‐CoV‐2 spike protein is common to all current COVID‐19 vaccines regardless of their mode of delivery (mRNA, protein or viral vectors). Therefore, the spike protein expressed in edible plant cells could potentially be used to develop cold chain‐free affordable booster vaccines for developing countries (Figure 1a‐g), in combination with any one of the currently available primary vaccines.

## Topical drug delivery using plant cells

The topical delivery of a plant‐derived antibody was first demonstrated for a secretory IgA/G against *Streptococcus mutans* surface antigen I/II, indicated for the prevention of dental caries (Ma *et al*., 1998). Other examples include the HIV‐neutralizing antibody 2G12 produced in tobacco (Ma *et al*., 2015) and Mapp66 produced in *N*. *benthamiana*, recognizing the receptor αCCR5 required for HIV‐1 attachment (Bogers *et al*., 2004). A monoclonal antibody against herpes simplex virus (HSV‐2) was produced in soybean and was able to diffuse into the vaginal mucus and prevent HSV‐2 infection in mice (Zeitlin *et al*., 1998). Topical application requires a high local dose of the antibody, and this can be readily achieved using the plastid expression system as shown for the antimicrobial peptides retrocyclin and protegrin. These products were expressed in tobacco plastids with yields of 38% and 26% of total soluble protein, respectively (Lee *et al*., 2011b). They were shown to kill *S. mutans* and prevent biofilm formation following a single topical application to a tooth mimetic surface (Liu *et al*., 2016) but their broader antimicrobial activity could make them useful against multiple bacterial and even viral pathogens, as confirmed by their ability to protect host tobacco plants against the plant pathogens *Erwinia carotovora* and tobacco mosaic virus.

## Molecular farming as a strategy to address endemic diseases

### Dengue fever

Dengue is an endemic disease of tropical and subtropical regions caused by one of four dengue virus serotypes (DENV‐1, DENV‐2, DENV‐3 and DENV‐4) carried mainly by the mosquitoes *Aedes aegypti* and *Aedes albopictus* (Khetarpal and Khanna, 2016; Pang *et al*., 2017;). Most infections are asymptomatic, but in 25% of cases viraemia may be present for 24–48 h before the onset of mild febrile dengue fever, which may progress in 0.5–5% of cases to severe and occasionally fatal dengue haemorrhagic fever and dengue shock syndrome (Yoshikawa *et al*., 2020). Dengue prevention mostly relies on vector control (Deng *et al*., 2020) but a live‐attenuated tetravalent vaccine is available (Dengvaxia) consisting of chimaeras of structural pre‐membrane and envelope genes of the four DENV serotypes combined with the non‐structural genes of the yellow fever 17D vaccine (Thomas and Yoon, 2019; Wilder‐Smith, 2020). At least six other vaccine formulations are under development (Pinheiro‐Michelsen *et al*., 2020).

As is the case for other endemic diseases that affect primarily developing countries, plants offer a promising platform for vaccine and antibody production because of the low costs, ease of distribution and the particular efficacy of VLP candidates. Recombinant proteins based on consensus envelope protein domain III (cEDIII) and non‐structural protein 1 (NS1) have been produced by transient expression in *N. benthamiana* (Kim *et al*., 2018; Marques *et al*., 2019; Pang *et al*., 2019) and in transgenic tobacco (Amaro *et al*., 2015; Gottschamel *et al*., 2016; Kim *et al*., 2010, 2015, 2017b;), lettuce (van Eerde *et al*., 2019; Kanagaraj *et al*., 2011; Maldaner *et al*., 2013), rice (Kim *et al*., 2013, 2014, 2016) and potato (Kim *et al*., 2017a). Transient expression produces soluble antigens with maximum yields of 600 mg/kg fresh leaf biomass (Martinez *et al*., 2010) although an HBcAg‐cEDIII fusion protein was mostly insoluble and the soluble fraction was not detected by Western blot (Pang *et al*., 2019). Transgenic plants produced lower yields than transient expression, achieving yields in the range 0.002–3.45% of total soluble protein (van Eerde *et al*., 2019; Kim *et al*., 2017a) or up to 18.5 mg/kg fresh leaf biomass (Kim *et al*., 2014).

The NS1 protein from DENV‐2 was expressed in tobacco as a diagnostic reagent for ELISA tests because it has a greater sensitivity than DENV‐1 with only a slightly lower specificity (Amaro *et al*., 2015; Marques *et al*., 2019;). The first dengue VLP vaccines were produced based on DENV‐3 polyprotein in lettuce chloroplasts (Kanagaraj *et al*., 2011) and cross‐reacted with the anti‐dengue primary polyclonal antibody. DENV‐1 VLPs produced in *N. benthamiana* by transient co‐expression of structural and non‐structural proteins were immunogenic in mice (Ponndorf *et al*., 2020). To improve oral tolerance, cEDIII was fused to CTB. The CTB–cEDIII fusion protein showed strong affinity for GM1 ganglioside (Kim *et al*., 2017a) and orally immunized mice generated high IgG and faecal IgA titres, and the sera reacted with all four DENV serotypes with similar potency (Kim *et al*., 2016). Without exogenous adjuvants, cEDIII fused to the binding epitope of the Ebola‐specific antibody 6D8 induced a virus‐neutralizing anti‐cEDIII humoral immune response in mice (Kim *et al*., 2015). A chimaeric antibody against the dengue EDII fusion loop (pE60) retained its antigen‐binding specificity and affinity compared to mE60 and its effective neutralizing activity against DENV‐2 and DENV‐4, but showed no antibody‐dependent enhancement in human K562 cells (Dent *et al*., 2016).

### West Nile fever

West Nile fever is caused by the neurotropic West Nile virus, which is primarily transmitted by mosquitoes (which acquire the virus from infected birds) but occasionally via infected blood or from mother to infant (Colpitts *et al*., 2012). The virus was discovered in Uganda in 1937, but has since spread to many parts of Europe, Asia and North America, where there are thousands of cases every year (Clark and Schaefer, 2020). Most infections are asymptomatic, but in about 20% of cases the infected individual suffers fever, headache, joint pains, vomiting, and diarrhoea, and ˜1% of cases can progress to severe encephalitis or meningitis (Bai *et al*., 2019; Rizzo *et al*., 2020;). The young and old are particularly at risk (Colpitts *et al*., 2012). The early phase of infection involves viral replication in dermal dendritic cells and keratinocytes, followed by a visceral organ dissemination phase and finally spreading to the central nervous system (Clark and Schaefer, 2020). West Nile virus also infects horses, and four veterinary vaccines are available (three inactivated and one live recombinant canarypox vaccine) but a vaccine has not yet been approved for humans (Kaiser and Barrett, 2019) although several have been tested in clinical trials (Ulbert, 2019).

Several West Nile vaccine candidates and antibodies based on domain III (DIII) of the envelope (E) protein have been expressed in plants, either by transient expression in tobacco (He *et al*., 2012; 2014a,b; Lai *et al*., 2010, 2012, 2014, 2018;) with a maximum yield of 8.1 g/kg fresh leaf biomass (Lai *et al*., 2010) or in transient lettuce with a maximum yield of 350 mg/kg fresh leaf biomass (He *et al*., 2012; Lai *et al*., 2012;). The recombinant DIII protein accumulated to the highest levels when directed to the endoplasmic reticulum rather than the plastids or cytosol and showed a potency equivalent to the same protein expressed in bacteria (He *et al*., 2014b). In a disease challenge experiment, the protein protected 100% of infected mice (Lai *et al*., 2018). Plant‐produced antibodies with different *N*‐linked glycan profiles retained the efficacy of the same antibodies produced in mammalian cells (He *et al*., 2014a; Lai *et al*., 2014;).

### Yellow fever

Yellow fever is another mosquito‐borne disease caused by a virus that infects humans and non‐human primates. It is endemic in Africa, where 90% of infections occur, but also in tropical/subtropical regions of South America and Asia, the Pacific and Australia, resulting in 80,000–200,000 infections per year, causing 30,000–60,000 deaths (Douam and Ploss, 2018). Yellow fever virus binds to glycosaminoglycan heparan sulphate on the surface of host cells, but the specific receptor that recognizes the major envelope protein (YFE) before virus fusion is unknown (Douam and Ploss, 2018). The virus initially replicates in the lymph nodes, particularly in dendritic cells, before invading the liver and infecting hepatocytes. Fatal cases reflect the loss of hepatocytes by apoptosis or necrosis and the resulting cytokine storm and shock (Quaresma *et al*., 2013). An efficacious yellow fever vaccine (YFV‐17D) is available based on a live‐attenuated strain (Barrett, 2017) and a single dose confers lifelong immunity that becomes effective within 30 days for 99% of recipients (Simon *et al*., 2020). The availability of an efficacious vaccine means that the molecular farming community has shown only limited interest in this disease. However, the YFE protein has been transiently expressed in tobacco, either alone or as a fusion with the bacterial enzyme lichenase (YFE‐LicKM), protecting up to 88% of immunized mice from lethal challenge and eliciting neutralizing antibodies in more than 80% of immunized non‐human primates (Tottey *et al*., 2018).

### Rabies

Rabies is an infection caused by lyssaviruses such as rabies virus, which is mainly transmitted by animal bites (Brunker and Mollentze, 2018; Rupprecht and Dietzschold, 2017). If an infection is identified promptly, rabies symptoms can be prevented by post‐exposure prophylaxis using injections of recombinant immunoglobulin (RIG) around the wound (Terryn *et al*., 2016). In the absence of treatment, the initial fever leads in almost all cases to lethal encephalitis, which kills 40,000–70,000 people per year, mostly in endemic areas of Asia, Central America, the Caribbean and North Africa (Jackson, 2016). Most cases recorded outside these areas involve migrants or travellers (Gautret *et al*., 2020).

As well as post‐exposure prophylaxis in humans, pre‐exposure vaccination of humans and potential animal reservoirs can help to reduce the spread of this disease. Rabies virus proteins and antibodies have been expressed in plants as a means to provide inexpensive reagents for developing countries to replace the very expensive human RIG and the less expensive but also less efficacious equine RIG. Examples of relevant proteins expressed in plants include rabies‐specific single‐chain antibodies and fusion proteins, the anti‐rabies monoclonal antibodies E559 and 62‐71‐3, a rabies glycoprotein fusion to the ricin toxin B chain (rgp‐rtxB), and the rabies virus surface glycoprotein and nucleoprotein, either as complete proteins or as epitopes. These have been expressed transiently in spinach and tobacco, as well as in transgenic maize, tobacco and tomato plants or cell lines (Girard *et al*., 2006; Loza‐Rubio *et al*., 2008; Singh *et al*., 2015; Tsekoa *et al*., 2016; Van Dolleweerd *et al*., 2014; Yusibov *et al*., 2002; **Table** **S1**).

The analysis of these plant‐derived rabies reagents has shown that they are functional and suitable for further development as therapeutics. For example, antibodies produced in tobacco cell suspension cultures were able to bind and neutralize the rabies virus *in vitro* (Girard *et al*., 2006). The rabies virus glycoprotein produced by transient expression in spinach using viral vectors was shown, after oral administration, to elicit significant antibody responses in three of five volunteers previously immunized against rabies with a conventional vaccine and five of nine volunteers who had not received a conventional vaccine (Yusibov *et al*., 2002). Furthermore, the glycoprotein of the Vnukovo rabies virus strain expressed in maize was orally administered to mice, inducing neutralizing antibodies and cross‐protecting against a challenge with a lethal rabies virus strain (Loza‐Rubio *et al*., 2008).

### Malaria

Malaria is an infectious disease caused by unicellular parasites of the genus *Plasmodium*, which are transmitted to humans and other mammals via mosquito bites (Talapko *et al*., 2019). The most severe form of malaria is caused by *P. falciparum*, which is often carried by female mosquitoes of the species *Anopheles gambiae*. This disease is endemic in Africa and parts of Asia, causing more than 400,000 deaths per year (WHO, 2020a). Infection of the human host commences when an infected mosquito takes a blood meal, causing motile parasites (sporozoites) to enter the blood stream. When these forms reach the liver, they invade hepatocytes and switch to an asexual reproductive form (merozoites) that proliferates, causing liver damage and infecting more red blood cells to perpetuate the asexual cycle. The damage caused during this process varies between individuals, in some cases causing asymptomatic parasitaemia but in others a range of symptoms ranging from mild fever to life‐threatening anaemia, metabolic acidosis and multi‐organ failure (Beales *et al*., 2000; Miller *et al*., 2002; Wassmer *et al*., 2015;). Some merozoites develop into gametocytes, which are taken up by mosquitoes to continue the parasitic life cycle.

Malaria can be treated with drugs such as chloroquine and artemisinin that inactivate the parasite, or can be prevented by interrupting the transmission cycle, for example by using mosquito nets and other vector control methods. However, these methods are only partially effective and parasite strains have evolved resistance to chloroquine, with emerging resistance to artemisinin also reported in some endemic regions. The extraction and purification of artemisinin from *Artemisia annua* are a very expensive process, but the oral delivery of artemisinin in the form of dried *A. annua* leaves has proven effective even against parasite strains resistant to artemisinin combination therapy and intravenous artesunate (Daddy *et al*., 2017).

The key goal in malaria prevention is a vaccine that covers multiple parasite strains and stages (pre‐erythrocytic/liver stage, erythrocytic stage and transmission‐blocking). A commercial vaccine (RTS,S/AS01, Mosquirix) based on the *P. falciparum* pre‐erythrocytic circumsporozoite protein (CSP) has been approved (efficacy = 30–40%) and is undergoing extended testing in Ghana, Kenya and Malawi as part of the WHO’s Malaria Vaccine Implementation Programme (WHO, 2020b). As for other diseases that disproportionately affect developing countries, the production costs of this vaccine are critical and plants offer a platform to produce multiple vaccines or vaccine cocktails while exploiting the economy of scale offered by greenhouse cultivation. Among the candidates that have been evaluated, *P*. *falciparum* surface proteins Pfs25, Pf38, Pfs230, PfGAP50, MSP19, MSP142 and AMA1, and the *P*. *vivax* merozoite surface protein MSP1 (as well as combinations thereof) have been expressed in tobacco, Arabidopsis, lettuce and rice (Beiss *et al*., 2015; Blagborough *et al*., 2016; Boes *et al*., 2015, 2016; Chen *et al*., 2016a; Lee *et al*., 2011a; Menzel *et al*., 2016; Voepel *et al*., 2014; **Table** **S1**). The maximum yield was 800 mg/kg fresh leaf biomass for the transient expression of Pfs230 in *N*. *benthamiana* (Farrance *et al*., 2011), and simple, cost‐effective heat‐based purification methods have been developed for some of the candidates (Buyel *et al*., 2014).

Oral immunization of BALB/c mice with *P*. *vivax* chimaeric recombinant MSP1/CSP induced antigen‐specific IgG1 production. The rabbit immune sera reacted well with the *P. falciparum* native protein and strongly inhibited the growth of blood‐stage parasites *in vitro* (Lee *et al*., 2011a). In another study, plasma from 31 semi‐immune African blood donors was assayed for reactivity against the antigens Pf38, AMA1 and MSP119 by ELISA. Positive reactivity was defined as a reading greater than twice the negative control value (Feller *et al*., 2013). Immunization of mice with one or two doses of Pfs25/coat protein VLPs plus the adjuvant Alhydrogel induced serum antibodies with complete transmission‐blocking activity throughout the 6‐month study period. The candidate vaccine (Pfs25‐FhCMB) was purified, characterized and evaluated for immunogenicity and efficacy using multiple adjuvants in a transgenic rodent model. Transmission‐blocking activity of ≤ 65% reduction in intensity and ≤ 54% reduction in prevalence was observed using Abisco‐100 adjuvant (Blagborough *et al*., 2016). Transmission‐blocking antibodies persisted for up to 6 months post‐immunization in mice and rabbits. Phase I testing revealed that the Pfs25‐FhCMB vaccine was safe in healthy volunteers, with no vaccine‐related serious adverse events and no evidence of any dose‐limiting or dose‐related toxicity. However, although Pfs25‐specific IgG was elicited in vaccinated patients in a dose‐dependent manner, the transmission‐reducing activity was weak, suggesting the need for an alternative adjuvant formulation (Chichester *et al*., 2018).

Malarial and cholera antigens have been fused together (CTB‐AMA1 and CTB‐MSP1) to develop dual cholera–malaria vaccines (Davoodi‐Semiromi *et al*., 2010). The yields obtained by plastid transformation were up to 600‐fold higher in tobacco and up to 400‐fold higher in lettuce compared to nuclear transformation. Increasing numbers of boosters with the malarial vaccine antigens increased the anti‐MSP1 antibody titres in immunized mice. Orally immunized mice generated both systemic and mucosal immune responses against the malarial antigens, but injected mice failed to generate IgA. Anti‐AMA1 antibodies in the serum of immunized mice bound to the *Plasmodium* schizont protein as an 83‐kDa polypeptide and also bound the apical end of the parasite at the ring stage as a further confirmation of specificity. The anti‐MSP1 antibodies in orally immunized mice bound to the *Plasmodium* ring and schizont proteins as a 190‐kDa polypeptide. Fluorescence‐labelled sera from immunized mice stained schizont stage parasites, further confirming the specificity of antibodies generated by oral vaccination. The ring stage was the predominant parasitic stage as determined by microscopy. The lowest parasitaemia was observed in the MSP119 group with the highest mean antibody titre. Relative inhibition of *Plasmodium* with the sera from vaccinated mice was equivalent to or better than the efficacy of the positive serum. The oral and injectable AMA1 vaccines conferred 102% and 105% inhibition in parasitemia assays, respectively.

### Tuberculosis

Tuberculosis is a disease primarily affecting the lungs, which is caused by the bacterium *Mycobacterium tuberculosis* (Fogel, 2015). Most cases are asymptomatic (latent tuberculosis) but about 10% progress to the active form of the disease, characterized by fever, chest pains and coughing, which is fatal in 50% of patients left untreated. Most of the 1.5–2 million deaths per year occur in developing countries, where it is more difficult to access the cocktail of first‐line antibiotics required for treatment (Fogel, 2015). The only approved vaccine against tuberculosis is the Bacillus Calmette–Guérin (BCG) vaccine developed in 1921. This is an attenuated vaccine derived from *M. bovis*, and when administered to children, it reduces the risk of infection by ˜20% and the risk of active disease by ˜60% (Cernuschi *et al*., 2018; Escobar *et al*., 2020). Other vaccines are under development, and as for other endemic diseases affecting developing countries, plants offer an inexpensive platform for the production of antigenic proteins or VLPs.

The development of subunit vaccines against tuberculosis has focused on *M. tuberculosis* secreted antigens such as ESAT‐6, Ag85B, Ag85A, MPT64, MPT83, CFP10 and dIFN. These antigens have been transiently expressed in tobacco and broccoli and stably expressed in tobacco, Arabidopsis, potato, lettuce and carrot (Dorokhov *et al*., 2007; Floss *et al*., 2010; Lakshmi *et al*., 2013; Permyakova *et al*., 2015; Rigano *et al*., 2006; Uvarova *et al*., 2013; Zelada *et al*., 2006; Zhang *et al*., 2012). High levels of *M. tuberculosis* antigens sufficient for purification have been produced by transient expression (Dorokhov *et al*., 2007; Floss *et al*., 2010; Zelada *et al*., 2006;), reaching 800 mg/kg fresh leaf biomass in tobacco (Dorokhov *et al*., 2007). In contrast, the stable expression of ESAT‐6, Ag85B, Ag85A, MPT64, MPT83, CFP10 and dIFN in potato leaves and carrot roots achieved yields of less than 0.01% of total soluble protein (Permyakova *et al*., 2015; Rigano *et al*., 2006; Uvarova *et al*., 2013; Zhang *et al*., 2012). Oral administration of antigens in mice has been shown to induce both cell‐mediated and humoral immunity (Floss *et al*., 2010; Kim *et al*., 2020; Permyakova *et al*., 2015; Saba *et al*., 2019, 2020; Uvarova *et al*., 2013; Zhang *et al*., 2012;).

### Helminth infections

Helminths are parasitic worms, including tapeworms, nematodes, schistosomes and filarial worms. More than one quarter of the world’s population is infected by one or more helminths, and such diseases are endemic in South America, sub‐Saharan Africa and South‐East Asia (WHO, 2020c). Helminths cause severe morbidity, including persistent diarrhoea and abdominal pain, and chronic infections in children can lead to malnutrition, impaired growth and delayed cognitive development. Helminth infections can be controlled using anthelmintic drugs, but overuse has led to the increasing prevalence of drug‐resistant worms in livestock and the same challenge could arise in humans (Sutherland and Leathwick, 2011). More than 100 vaccine trials have been carried out with 80 different recombinant antigens targeting 22 helminth species (Geldhof *et al*., 2007) but few have demonstrated efficacy in challenge studies, possibly due to the presence of complex glycan structures that are difficult to mimic in recombinant proteins (Hokke and van Diepen, 2017).

The first helminth vaccine candidates expressed in transgenic plants were *Schistosoma japonicum* ferritin in alfalfa (Yuan *et al*., 2008), *Fasciola hepatica* FH3 antigen in rapeseed (Li *et al*., 2003) and *Ascaris suum* As16 antigen fused to CTB in rice (Matsumoto *et al*., 2008). More recently, transient expression in *N*. *benthamiana* has become the dominant platform. For example, the aspartic protease‐1 antigen from the human hookworm *Necator americanus* (Na‐APR‐1 (M74)) was expressed at high levels in *N. benthamiana* (up to 300 mg/kg fresh leaf biomass), exceeding the yields achieved in the yeast *Pichia pastoris* (Seid *et al*., 2015). Na‐APR‐1 was directed to the secretory pathway using the tobacco PR1a signal peptide and the construct also included a KDEL sequence for retrieval to the endoplasmic reticulum and a His_6_ tag for protein purification. This plant‐derived Na‐APR‐1 is now part of a bivalent human hookworm vaccine developed by the Sabin Vaccine Institute Product Development Partnership and has entered phase I clinical trials (Hotez *et al*., 2016). *N*. *benthamiana* has also been used to produce glycoproteins from the human blood fluke *Schistosoma mansoni*, although not directly for vaccine purposes (Wilbers *et al*., 2017). The glycoproteins omega‐1 and kappa‐5 were targeted to the secretory pathway using an Arabidopsis chitinase signal peptide, causing ˜90% of the protein to reach the apoplast. This highly efficient secretion allowed product recovery by apoplast fluid extraction, a non‐destructive single‐step purification method yielding > 0.5 mg of omega‐1 or kappa‐5 per plant (each plant produces 3–4 g of fresh leaf material). This study showed that the native helminth glycan structures (Lewis X and LDN‐F) could be synthesized on omega‐1 and kappa‐5 by fine‐tuning the co‐expression of glycosyltransferases. Although *N. benthamiana* provides an efficient platform for the expression of glyco‐engineered proteins, the diverse and complex glycan structures of helminths are poorly understood. Here, also plants can offer a helping hand, as shown by the functional characterization of 10 *S*. *mansoni* fucosyltransferases by transient expression in *N. benthamiana*, allowing the synthesis of the helminth glycan motif F‐LDN‐F (van Noort *et al*., 2020).

## Neglected and re‐emerging diseases

### Cholera

Cholera is an acute bacterial disease caused by serotypes O1 and O139 of *Vibrio cholerae*, which infect the intestine and cause severe diarrhoea and dehydration. The historical prevalence of the disease has been reduced by improvements in water quality, sanitation and hygiene, and the development of oral and intravenous rehydration therapy has reduced morbidity and mortality. However, cholera still causes up to 100,000 deaths every year, mostly in cholera‐endemic developing countries. Vaccines against cholera are designed to provide intestinal mucosal immunity; hence, the most widely used vaccines are orally delivered inactivated or live‐attenuated bacterial strains (Clemens *et al*., 2017). These elicit antibodies against the bacterial lipopolysaccharide and also the CTB subunit. The latter is secreted by the bacteria when they penetrate the intestinal mucosa and form a biofilm. The hexameric toxin, comprising five CTB subunits and the cholera toxin A (CTA) subunit, enters cells and causes the secretion of water and electrolytes into the intestinal lumen, thus triggering the disease symptoms. Antibodies against the bacterium and its toxin work synergistically to prevent infection (Lange and Holmgren, 1978). Inactivated oral vaccines are effective against both epidemic and endemic cholera, whereas live‐attenuated vaccines are unsuitable for endemic settings but useful for travellers (Chen *et al*., 2016b; Richie *et al*., 2000). Inactivated oral cholera vaccines also confer herd immunity (Ali *et al*., 2005; Longini *et al*., 2007).

Interest in the production of cholera vaccines in plants reflects the opportunity to present the vaccine antigens either in fresh edible tissues such as fruits and leaves, or in dry and inert edible tissues such as seeds, if the dose and moisture content can be controlled. In both cases, oral vaccination can be achieved simply by the consumption of vaccine‐containing foods after minimal preparation, and the seeds also allow the storage and distribution of vaccines in remote areas without a cold chain. In the fresh tissue category, CTB has been expressed in tomato fruits (Jani *et al*., 2002) and in the leaves of *N. benthamiana* plants (Hamorsky *et al*., 2013, 2015), the latter achieving remarkable yields of ˜1 g/kg fresh leaf biomass (transgenic plants) and ˜3 g/kg fresh leaf biomass (transient expression). These experiments confirmed that recombinant CTB forms a pentamer and interacts with GM1 ganglioside like the native toxin and that both glycan‐free and glycosylated versions raise protective antibodies in mice. The production of cholera vaccines in dry seeds has been pursued by Hiroshi Kiyono’s group at the University of Tokyo, which has published a series of articles concerning the expression of CTB in transgenic rice seeds, named MucoRice‐CTB. These studies have shown that CTB remains stable in seeds for more than 3 years and elicits both secretory IgA (mucosal response) and IgG (systemic response) after oral administration in mice (Yuki and Kiyono, 2008) and macaques (Nochi *et al*., 2009). Interestingly, the inclusion of CTA did not broaden the immune response—antibodies were elicited against CTB but not CTA or native rice proteins (Yuki *et al*., 2009). The group also found that CTB was processed *in planta* into two forms that assembled into heterocomplexes. Accordingly, they mutated an *N*‐linked glycan acceptor site to produce a uniform vaccine (MucoRice‐CTB/Q) which elicited an immune response similar to that of the original formulation in mice and macaques (Yuki *et al*., 2013). Although one of the challenges involved in the production of edible plant tissues containing vaccine antigens is the control of yields to ensure consistent dosing, the MucoRice‐CTB content of rice endosperm was consistent across multiple harvests of rice plants grown in a closed hydroponic system due to the carefully controlled environmental conditions (Kashima *et al*., 2016).

Davood‐Semiromi *et al*., (2010) reported the longest cholera vaccine study using plant‐derived vaccines. Animals were boosted until 267 days with CTB expressed in tobacco or lettuce chloroplasts and were challenged on day 303. Protection against cholera toxin challenge was observed following oral immunization (100%) or subcutaneous immunization, and protection correlated well with CTB‐specific titres of intestinal IgA and serum IgG1 in orally immunized mice, but only IgG1 was detected in subcutaneously injected mice. Multiple oral boosters conferred durable immunity, lasting more than half of the mouse life span. With the current cholera vaccine, immunity is lost in children within 3 years and adults are not fully protected.

### Measles

Measles is a highly contagious viral disease that causes more than 100,000 deaths every year, with 1.5–2.9% fatality based on computer models (Portnoy *et al*., 2019). Following the onset of fever, cough and runny nose, the characteristic symptom is a rash covering the entire body. In some cases, the virus induces immunosuppression, leading to complications such as diarrhoea, ear infections and pneumonia. Less common complications include seizures, blindness and encephalopathy. Measles can be prevented by vaccination, which involves the administration of an attenuated wild‐type strain that elicits the production of neutralizing antibodies (Griffin, 2018). Herd immunity is achieved by administering two doses of the vaccine to > 90% of the population at 9–15 months and 3–4 years of age, often combined with the vaccines for mumps, rubella and in some cases also chickenpox (Moss, 2017). This has led to the effective control of the disease in most developed countries, with occasional spikes resulting from vaccine hesitancy (Larson *et al*., 2014).

As discussed above for cholera, interest in the use of plants for the production of measles virus is primarily driven by the potential of an oral vaccine (Muller *et al*., 2003). All studies published thus far have involved the expression of the immunodominant measles virus hemagglutinin (MVH) protein or an epitope derived therefrom and have examined the immunogenicity of the antigen either purified and injected or delivered orally in plant tissue. The first studies expressed MVH in transgenic tobacco and found that the recombinant antigen was immunogenic in mice both orally and following intraperitoneal injection (Huang *et al*., 2001) and that the mucosal (secretory IgA) and systemic (IgG) immune response was stronger if a single dose of a DNA vaccine was followed by multiple oral boosters of tobacco leaves containing the antigen (Webster *et al*., 2002, 2005). The same group reported similar results when the antigen was expressed in lettuce and administered via the nasal route (Webster *et al*., 2006). MVH is one of a small number of vaccines to be expressed in carrot, and the recombinant antigen was shown to generate high titres of neutralizing antibodies when leaf or root extracts were injected into mice (Marquet‐Blouin *et al*., 2003). The same group later studied a range of epitopes based on the MVH protein, including the [L4T4]2 polyepitope combining 2 × 4 repeats of the loop‐forming hemagglutinin noose epitope (L, residues 386–400) and the tetanus toxoid T‐cell epitope (T, residues 830–844) (Bouche *et al*., 2003, 2005). Interestingly, although the L epitope was derived from a single isolate, the immune response was sufficient to neutralize all field isolates of the virus, suggesting that the polyepitope adopted multiple conformations to induce a diverse repertoire of B cells.

### Crimean‐Congo haemorrhagic fever

Crimean‐Congo haemorrhagic fever (CCHF) is the most important tick‐borne viral disease in humans, causing sporadic cases or outbreaks of severe illness across a huge geographic area, from western China to the Middle East and south‐eastern Europe and throughout most of Africa, with fatality rates of 5–80% (Blair *et al*., 2019). The virus responsible for this disease (CCHFV) is carried by many species of ticks, including at least 30 from the genera *Haemaphysalis* and *Hyalomma*. Most infections are directly transmitted by tick bites, but transmission also occurs by contact with infected domestic animals/livestock and other humans. The eradication of ticks that carry CCHFV has proven inefficient and expensive because infected domestic animals are often asymptomatic and act as disease reservoirs following tick repopulation (Mendoza *et al*., 2018). Nosocomial outbreaks of CCHFV occur in endemic areas and are difficult to detect because the non‐specific flu‐like symptoms lead to late recognition and diagnosis (Fletcher *et al*., 2017). There are no specific treatment options for CCHF, and the only effective vaccine is an inactivated formulation developed in the 1960s by Soviet researchers, which was licensed in Bulgaria in 1974 for use in endemic regions (Keshtkar‐Jahromi *et al*., 2011). Although safety and efficacy data for the vaccine have not been established in controlled trials, it has a good track record based on the decline in reported cases and can induce low levels of CCHFV‐neutralizing antibodies after multiple doses (Mousavi‐Jazi *et al*., 2012). However, the crude nature of vaccine preparation makes it unlikely to be adopted more widely (Buttigieg *et al*., 2014). A more effective and refined vaccine is therefore required now that the WHO has added CCHF to its Blueprint List of Priority Diseases in second position after COVID‐19.

Interest in the use of plants for the production of CCHF vaccines primarily reflects the opportunities presented by the ability to grow plants locally so that vaccines can be made available in rapid response to an outbreak of the disease. It is also important to note that the oral vaccination of animals by feeding them on plants expressing CCHFV components may be an effective strategy to eliminate disease reservoirs and therefore increase the efficacy of tick elimination programmes. Like other *Bunyaviridae*, CCHFV has a tripartite RNA genome encoding two external glycoproteins (G_N_, G_C_) produced by the proteolytic cleavage of a precursor, a nucleocapsid protein (N) and a large transcriptase (L). Both the glycoprotein and nucleocapsid protein have been expressed in plants and evaluated as vaccine candidates. The glycoprotein was expressed in tobacco leaves and hairy roots, with yields of 1.4 and 1.8 g/kg fresh biomass, respectively (Ghiasi *et al*., 2011). Mice were fed on the leaves or roots (fed group) with or without a subcutaneous injection of the same plant‐derived vaccine candidate (fed‐boost group) and these were compared to controls injected with the attenuated CCHFV vaccine (positive control) and an untreated negative control. All the immunized mice showed a serum response (IgG) as well as a mucosal response (IgA) with the highest titres of both antibodies in the fed‐boost group (Ghiasi *et al*., 2011). The CCHFV nucleocapsid protein has also been expressed in plants (*N. benthamiana*) and cross‐reacted with anti‐CCHFV IgG in sera from convalescent patients, but its immunogenicity has not been evaluated directly (Atkinson *et al*., 2016).

### Polio

Polio (poliomyelitis) is an infectious disease caused by poliovirus, which is spread via the oral‐faecal route. Most cases occur without symptoms, but in a minority (˜0.5%), there is a period of muscle weakness often but not exclusively affecting the legs. Most people recover, but up to 5% of children and 30% of adults within this group can progress to a more serious and often fatal disease stage. The disease is preventable by vaccination with multiple doses of inactivated or live‐attenuated virus. Coordinated global vaccination programmes have led to the near eradication of poliovirus types 2 and 3 and wild‐type polio infections were reduced to just 46 cases in 2016, rising to 176 in 2019 (mainly in Pakistan and Afghanistan). However, both vaccines have drawbacks such as the need to cultivate live virus, the risk of vaccine‐derived poliomyelitis (365 cases in 2019) and production bottlenecks. Because these drawbacks prevent the total global eradication of polio, a virus‐free vaccine is of interest for the WHO polio eradication programme. Since 2013, the WHO has funded an ongoing project for the development of a VLP polio vaccine. The consortium that works on this project includes eight members using different expression platforms and analysis methods to achieve the main goal of the project, the production of antigenic VLPs of all three polio serotypes. However, the development of polio VLPs has been hampered because, in the absence of the genomic RNA, the virus capsid is antigenically unstable, rapidly converting to the C form which does not stimulate protective immunity. To develop antigenically stable VLPs, predominantly in the D form, the National Institute for Biological Standards and Control (NIBSC) selected thermostable empty capsids representing all three serotypes (Fox *et al*., 2017), which are now used to produce polio VLPs in different expression systems.

To produce VLPs in plants, the P1 capsid precursor region from each of the stabilized mutants was transiently expressed in *N. benthamiana* in the presence of the poliovirus 3CD protease to produce the mature capsid proteins (Marsian *et al*., 2017). The plant‐derived stabilized VLPs representing serotype 3 were comparable to the standard inactivated polio vaccine in terms of immunogenicity when tested in a mouse model expressing the poliovirus receptor. Similar results were found for polio serotype 1 (unpublished) and work is ongoing to produce VLPs representing serotype 2 (which have proven more difficult to generate regardless of the expression platform). The yield of stabilized VLPs representing serotypes 1 and 3 produced in the *N. benthamiana* transient expression platform was equivalent to or better than that achieved in yeast, mammalian cells, baculovirus‐infected insect cells and a cell‐free expression system.

Although a mucosal vaccine against poliovirus is highly desirable, the Sabin Vaccine Institute’s live‐attenuated vaccine (OPV2) was withdrawn by the WHO due to circulating vaccine‐derived polioviruses and polio virus transmission (Hird and Grassly, 2012; Minor, 2009; Yarri *et al*., 2013). The Daniell laboratory has developed a virus‐free oral booster vaccine that does not require a cold chain by expressing the vaccine antigen VP1 common to all poliovirus serotypes in chloroplasts. The oral delivery of lyophilized plant cells expressing CTB‐VP1 induced strong mucosal and systemic immunity, with high antigen‐specific titres of both IgG1 and IgA. Poliovirus neutralization studies using sera from immunized animals confirmed protection against all three poliovirus serotypes, with high‐level neutralization titres and seropositivity (Chan *et al*., 2016). The first study used 15 booster doses over 400 days (Xiao and Daniell, 2017) whereas subsequent studies reduced the number of boosters to three doses over 300 days and explored the efficacy of oral plant‐derived adjuvants (saponin or squalene) and antimicrobial peptides (LL37 and PG1) to enhance immune response (Daniell *et al*., 2019b). Long‐term maintenance of IgG1 and IgA titres and protection against all three poliovirus serotypes were achieved with only three boosters.

## The future of molecular farming for endemic diseases

Molecular farming has received substantial recent press coverage focusing on its potential to satisfy the demand for vaccines, research reagents and therapeutics in the face of a rapidly spreading epidemic or pandemic diseases. Such diseases threaten to overwhelm traditional production platforms and the extra production capacity offered by plants would be welcome. However, the COVID‐19 pandemic has also brought to light major deficiencies, including the cost implications of global vaccination programmes and the need for a cold chain. More than 130 countries have yet to receive a single dose of COVID‐19 vaccine while wealthy countries have stockpiled available doses. When the WHO withdrew the oral polio vaccine in 2015 due to vaccine‐derived poliovirus infections, most developing countries could afford a single intravenous vaccine but not a booster. Therefore, the COVID‐19 pandemic is not the only example of an inadequate vaccine supply and affordability issues. Plant‐derived oral booster vaccines offer a timely and affordable solution that eliminates the cold chain requirement.

Oral vaccines are particularly valuable as a means to tackle endemic diseases, which are the scourge of healthcare systems particularly in developing countries (Rybicki *et al*., 2013). Such diseases have persisted for decades and often lead to epidemic outbreaks when cases suddenly spike in a particular region. The current focus on responses to epidemics and pandemics has drawn attention from perhaps the most enduring benefits of molecular farming, namely the ability to grow plants in local regions to allow the inexpensive production of drugs and vaccines for local healthcare systems. Such local production facilities could be paired with portable downstream processing suites when the product is intended for purification, but the oral or topical application of plant tissues or crude extracts releases plants from the demands of extensive product purification, in some cases allowing the consumption of medicines as unprocessed or part‐processed edible tissues (Daniell *et al*., 2019a, 2021; Ma *et al*., 2013).

We have considered the potential of molecular farming to address endemic and re‐emerging diseases, particularly when the drugs can be manufactured, stored and distributed in remote areas without a cold chain. In such cases, the important aspects of molecular farming are not the speed of production, but the scalability, ease of integration into local cultivation and distribution systems, and the development of innovative strategies to achieve both local (mucosal) and systemic immunity through the delivery of recombinant proteins presented in plant tissues. Another important issue that is particularly relevant for the treatment and prevention of endemic diseases in developing regions is to ensure that the technology and product can be made available to local stakeholders without the encumbrance of IP restrictions and onerous licensing requirements, therefore allowing these valuable medicines to be delivered to those most in need (Drake and Thangaraj, 2010). Even so, it is clear that much of the effort thus far has been directed towards the optimization of gene expression in plants to demonstrate the principle of molecular farming, with less focus on clinical development. Historically, the path to the clinic has been obstructed by the unwillingness of pharmaceutical companies to transition from established platform technologies and the lack of a regulatory framework, but this is no longer the case. The COVID‐19 pandemic has shown that new technologies can be embraced and developed quickly, with the Moderna vaccine as a key example, and the plant‐based subunit and VLP vaccines developed by companies such as Medicago as another, specifically related to molecular farming. The slow progress towards clinical development for molecular farming may reflect the lack of production facilities, although several construction projects around the world are underway to address this (Fischer and Buyel, 2020). In the case of orally administered products, progress has also been slowed by the challenge of achieving precise antigen doses (Kashima *et al*., 2016), and the perceived risk that pharmaceutical products could end up in the food chain (Spok *et al*., 2008). However, the clinical advancement of antigens produced in edible leaves will be encouraged by recent progress in the high‐level expression of antigens, the accurate control of antigen doses, the removal of antibiotic resistance genes, the large‐scale production of leaves in FDA‐approved hydroponic growth facilities, the tightly controlled removal of moisture, batch‐to‐batch records for regulatory compliance and strict bioburden validation of microbes on the leaf surface accomplished recently (Daniell *et al*., 2019a, 2020, 2021; Srinivasan *et al*., 2021; Su *et al*., 2015;).

## Conflicts of interest

The authors declare no conflicts of interest.

## Author contributions

PC and TC conceived the topic. All authors contributed sections to this article based on their expertise and experience, provided comments and recommendations on the combined draft and approved the final version.

## Supporting information

**Table S1** Classification of endemic and re‐emerging diseases based on their epidemiology.
