# Supplementary material for: Contributions of the international plant science community to the fight against infectious diseases in humans—part 2: Affordable drugs in edible plants for endemic and re‐emerging diseases
Source: Plant Biotechnol J. 2021 Jul 19;19(10):1921–36. doi: 10.1111/pbi.13658 (PMC8486237; doi:10.1111/pbi.13658)
Supplement: Supplementary file 1 — Table S1 Classification of endemic and re‐emerging diseases based on their epidemiology. [file PBI-19-1921-s001.docx]

**Supplementary files**

Supplementary Table 1. Classification of endemic and re-emerging diseases based on their epidemiology.

| **Disease** | **Prevalence** | **Current treatments** | **Vaccine available?** |
| --- | --- | --- | --- |
| Dengue fever | In 2000, 505,430 cases of dengue were reported worldwide. The mortality rate was a 0.2% (960 deaths). Currently, dengue is endemic in more than 100 countries worldwide. In 2019, 4.2 million of cases of dengue were reported, an 8-fold increase compared to 2000. The highest number of cases (3.1 million) was reported in Brazil and Ecuador, followed by the Philippines (420,000 cases) and Vietnam (320,000 cases) [1]. | Treat symptoms (paracetamol administration) [2] | Yes [3] |
| West Nile fever | West Nile virus (WNV) is endemic in Asia, Africa, and the Middle East. WNV was first identified in Uganda, in 1937. A number of outbreaks occurred in Africa from the 1950s to the 1980s. In 1950, a serological study in Cairo demonstrated that WNV is widespread in the population, mostly in children. Another seropositivity study in Egypt demonstrated that WNV is endemic in this region. In 1951, there were 417 serologically confirmed WNV cases in Israel with a fatality rate of 8.3% (35 deaths), and every subsequent summer has seen outbreaks of different sizes. In 1996, there was a WNV outbreak in Romania, with 343 positive cases reported and a mortality of 5% (17 deaths). In 1999, a WNV outbreak occurred in Russia, with 183 reported cases and a mortality of 21.9% (40 deaths) [4]. In 1999, it was introduced to the USA [5]. In 2018, 2647 infections were reported in the USA, with a mortality of 6.2% (165 deaths) [6]. | Treat symptoms (headaches control, antiemetic therapy and rehydration) [7] | No |
| Yellow fever | Yellow fever is endemic in 33 countries in Africa and 11 countries in South America [8]. In 1961, more than 100,000 yellow fever cases were reported in Ethiopia with 30,000 deaths. From 1986 to 1994 there was a major yellow fever outbreak in Nigeria, and 20,495 cases were reported with a mortality rate of 30% [9]. A modeling study estimated that in 2013 in Africa there were 84,000-170,000 severe cases of yellow fever with 29,000-60,000 deaths [8]. From December 2015 to October 2016, 884 cases were reported with 121 deaths. From December 2016 to May 2017, 792 cases were reported with a mortality rate of 35%. A further 723 cases and 274 deaths were reported until February 2018 [9]. | Supportive treatment (hydration, treat liver and kidney failure and fever) and antibiotics (if bacteria-associated infections) [8] | Yes [8,9] |
| Rabies | Rabies is endemic worldwide [10]. It is estimated that rabies causes 59,000 human deaths each year in 150 countries, 95% of the cases occur in Asia and Africa. In Asia, the estimated human deaths per year is 35,152, and in Africa is 21,476 [11]. The incidence of rabies 2.33 cases per 100,000 humans was estimated [12]. | Rabies vaccine, dose of human rabies immune globulin and wound treatment [13,14] | Yes [15–17] |
| Malaria | Malaria mostly occurs in poor, tropical and subtropical areas. Africa is the most affected region, with 94% of all malaria deaths [18]. In 2019, 229 million cases of malaria were estimated worldwide, with 0.18% mortality (409,000 deaths). The global incidence of malaria decreased by 25% from 2010 to 2016. | Oral antimalarials (if uncomplicated malaria) or parenteral antimalarial therapy (if severe malaria) [20] | Yes [21,22] |
| Tuberculosis | In 2018, 7,600,000 cases of tuberculosis were reported, many in China (1,430,000 cases), India (1,350,000 cases), and Indonesia (268,000 cases). A small, but increasing number of multidrug-resistant tuberculosis cases has been recorded in recent years [23]. | Antibiotics [24] | Yes [23,25] |
| **Re-emerging or rare/neglected diseases** | | | |
| Cholera | In 2016, 131,121 cases of cholera were reported worldwide, with a mortality of 1.8% (2,420 deaths). Africa reported 54% of the cases, followed by Hispaniola (32%) and Asia (13%) [26]. In 2017, 71 countries reported 1,227,391 cholera cases with a mortality of 0.5% (5,654 deaths). Yemen reported 84% of all cases and 41% of all deaths [27]. | Rehydration solution [28] | Yes [29] |
| Measles | In 2000, there were 145 cases of measles per million people with an estimated 550,000 deaths. This was reduced after the first dose of measles vaccine, and the incidence in 2016 was 19 cases per one million people with 89,780 deaths [30]. In 2014-2015, the estimated global measles cases were 9.7 million with 134,200 deaths, most of the cases were reported in the African region (98,621 cases) followed by the Western Pacific Region (65,176 cases) and the South-East Asian region (29,109 cases) [31]. In 2019, the cases had risen by 300% compared to 2018 in regions with low vaccination rates (some parts of Europe and the USA) [30] | Supportive and symptom treatments and vitamin A treatment (if children severe measles) [32] | Yes [33-35] |
| Crimean-Congo hemorrhagic fever (CCHF) | From 1953 to 2010, 6000 cases of CGHF were reported in south eastern Europe [36]. Until 2016, the mortality rate was 32.4%. More than 1000 cases are reported each year in Turkey and south eastern Europe [37]. | Ribavirin therapy [38] | Yes [39] |

**References**

1. WHO (2020) Dengue and severe dengue. <https://www.who.int/news-room/fact-sheets/detail/dengue-and-severe-dengue>.

2. Rajapakse S, Rodrigo C, Rajapakse A (2012) Treatment of dengue fever. Infect Drug Resist 5:103–112.

3. Biswal S, Reynales H, Llorens XS, Lopez P, Tabora CB, Kosalaraksa P, Sirivichayakul C, Watanaveeradej V, Rivera L, Espinoza F, Fernando LK, Dietze R, Luz K, Da Cunha RV, Jimeno J, Medina EL, Borkowski A, Brose M, Rauscher M, LeFevre I, Bizjajeva S, Bravo L, Wallace D (2019) Efficacy of a tetravalent dengue vaccine in healthy children and adolescents. N Engl J Med 381:2009–2019.

4. Chancey C, Grinev A, Volkova E, Rios M (2015) The global ecology and epidemiology of West Nile Virus. Biomed Res Int 2015.

5. Medscape (2020) Salinas JD, Steiner ML: West Nile Virus: Practice Essentials, Pathophysiology, Epidemiology. <https://emedicine.medscape.com/article/312210-overview#a6>.

6. CDC (2020) Final Cumulative Maps and Data | West Nile Virus. <https://www.cdc.gov/westnile/statsmaps/cumMapsData.html>.

7. CDC (2018) West Nile virus disease therapeutics: review of the literature for healthcare providers. doi:10.1016/B978-1-4377-0126-5.00091-4.

8. WHO (2019) Yellow fever. <https://www.who.int/news-room/fact-sheets/detail/yellow-fever>.

9. Douam F, Ploss A (2018) Yellow fever virus: Knowledge gaps impeding the fight against an old foe. Trends Microbiol 26:913–928.

10. PAHO (2017) Zoonoses. <https://www.paho.org/salud-en-las-americas-2017/?tag=rabies>.

11. WHO (2020) Epidemiology and burden of disease. <https://www.who.int/rabies/epidemiology/en/>.

12. Jemberu WT, Molla W, Almaw G, Alemu S (2013) Incidence of Rabies in Humans and Domestic Animals and People’s Awareness in North Gondar Zone, Ethiopia. PLoS Negl Trop Dis 7.

13. WHO (2020) Rabies. Treatment. <https://www.who.int/rabies/about/home_treatment/en/>.

14. CDC (2019) Rabies Postexposure Prophylaxis (PEP) | Medical Care | Rabies. <https://www.cdc.gov/rabies/medical_care/index.html>.

15. CDC (2020) Vaccine Information Statement | Rabies | VIS. <https://www.cdc.gov/vaccines/hcp/vis/vis-statements/rabies.html>.

16. CDC (2014) Medical Care: Rabies Vaccine - Rabies. <https://www.cdc.gov/rabies/medical_care/vaccine.html>.

17. NHS (2020) Rabies - Vaccination - NHS. <https://www.nhs.uk/conditions/rabies/vaccination/>.

18. CDC (2021) Malaria - Malaria Worldwide - Impact of Malaria. <https://www.cdc.gov/malaria/malaria_worldwide/impact.html>.

19. WHO (2018) Malaria. <https://www.who.int/data/gho/data/themes/malaria>.

20. CDC (2020) Treatment of Malaria (Guidelines For Clinicians). doi:10.1016/S0140-6736(05)66420-3.

21. Science | AAAS (2019) First malaria vaccine rolled out in Africa—despite limited efficacy and nagging safety concerns. <https://www.sciencemag.org/news/2019/11/first-malaria-vaccine-rolled-out-africa-despite-limited-efficacy-and-nagging-safety>.

22. Raj DK, Das Mohapatra A, Jnawali A, Zuromski J, Jha A, Cham-Kpu G, Sherman B, Rudlaff RM, Nixon CE, Hilton N, Oleinikov AV, Chesnokov, O, Merritt J, Pond-Tor S, Burns L, Jolly G, Ben Mamoun C, Kabyemela E, Muehlenbachs A, Lambert L, Orr-Gonzalez S, Gnädig NF, Fidock DA, Park S, Dvorin JD, Pardi N, Weissman D, Mui BL, Tam YK, Friedman JF, Duffy PE, Kurtis JD (2020) Anti-PfGARP activates programmed cell death of parasites and reduces severe malaria. Nature 582:104-108.

23. WHO (2019) Global Tuberculosis Report. <https://www.who.int/teams/global-tuberculosis-programme/tb-reports>.

24. NHS (2019) Tuberculosis (TB) - Treatment. <https://www.nhs.uk/conditions/tuberculosis-tb/treatment/>.

25. Montagnani C, Chiappini E, Galli L, de Martino M (2014) Vaccine against tuberculosis: What’s new? BMC Infect Dis 14:1–9.

26. WHO (2017) Cholera. <https://www.who.int/data/gho/data/themes/cholera>.

27. WHO (2018) Weekly Epidemiological Record, 21 September 2018. Wkly Epidemiol Rec 93:489–500.

28. Pietroni MAC (2020) Case management of cholera. Vaccine 38:A105–A109.

29. WHO (2010) Cholera vaccines: WHO position paper.

30. UpToDate (2020) Measles: Epidemiology and transmission. <https://www.uptodate.com/contents/measles-epidemiology-and-transmission>.

31. Coughlin MM, Beck AS, Bankamp B, Rota PA (2017) Perspective on global measles epidemiology and control and the role of novel vaccination strategies. Viruses 9.

32. CDC (2020) For Healthcare Professionals - Diagnosing and Treating Measles. <https://www.cdc.gov/measles/hcp/index.html>.

33. WHO (2010) Cholera vaccines: WHO position paper-Recommendations. Vaccine 28:4687-8.

34. Richardus R, Alam K, Kundu K, Chandra Roy J, Zafar T, Chowdhury AS, Nieboer D, Faber R, Butlin CR, Geluk A, Richardus JH (2019) Effectiveness of single-dose rifampicin after BCG vaccination to prevent leprosy in close contacts of patients with newly diagnosed leprosy: A cluster randomized controlled trial. Int J Infect Dis 88:65-72

35. Gans H, Maldonado YA (2020). Measles: Epidemiology and transmission. UpToDate

36. Mertens M, Schmidt K, Ozkul A, Groschup MH (2013) The impact of Crimean-Congo hemorrhagic fever virus on public health. Antiviral Res 98:248–260.

37. Tsergouli K, Karampatakis T, Haidich AB, Metallidis S, Papa A (2020) Nosocomial infections caused by Crimean–Congo haemorrhagic fever virus. J Hosp Infect 105:43–52.

38. Arab-Bafrani Z, Jabbari A, Mostakhdem Hashemi M, Arabzadeh AM, Gilanipour A, Mousavi E (2019) Identification of the crucial parameters regarding the efficacy of ribavirin therapy in Crimean-Congo haemorrhagic fever (CCHF) patients: A systematic review and meta-analysis. J Antimicrob Chemother 74:3432–3439.

39. Keshtkar-Jahromi M, Kuhn JH, Christova I, Bradfute SB, Jahrling PB, Bavari S (2011) Crimean-Congo hemorrhagic fever: Current and future prospects of vaccines and therapies. Antiviral Res 90:85–92.
